# Force plate assessments in reconnaissance marine training company

**DOI:** 10.1186/s13102-023-00796-z

**Published:** 2024-01-13

**Authors:** Trevor Barrett, Robert Faulk, Army Master Sergeant, Jill Boberg, Matthew Bartels, Marine Lieutenant Colonel, Leslie A. Saxon

**Affiliations:** University of Southern California Institute for Creative Technologies, Center for Body Computing, Los Angeles, CA United States

**Keywords:** Marine training, Forceplates, Training load

## Abstract

The ability to obtain dynamic movement assessments using force plate technology holds the promise of providing more detailed knowledge of the strength, balance and forces generated by active-duty military personnel. To date, there are not well-defined use cases for implementation of force plate assessments in military training environments. We sought to determine if force plate technology assessments could provide additional insights, related to the likelihood of graduation, beyond that provided by traditional physical fitness tests (PFT’s), in an elite Marine training school. Serial force plate measures were also obtained on those Marines successfully completing training to determine if consistent measures reflecting the effects of training on muscle skeletal load-over-time could be accurately measured. A pre-training force plate assessment performed in 112 Marines did not predict graduation rates. For Marines who successfully completed the course, serial measures obtained throughout training were highly variable for each individual and no firm conclusions could be drawn related to load imposed or the fitness attained during training.

## Introduction

Movement assessments, evaluated using force plate technology, are now available and utilized by diverse military units [1]. There is the potential to understand individual movement characteristics and vulnerabilities to injury with the data, which provide a kinetic assessment of movement by measuring movement-related ground forces [2]. Obtaining serial measures of a service member on a force plate, over time, can also provide comparative data and help understand how military training or operational duties impact an individual [1–3]. However, there is little published literature that describes how force plate assessments can identify a new capability such as injury prevention or augment a current capability such as fitness training or recovery from injury in active-duty service members. There are important differences between military and athletic training to consider when assessing the utility of technologies like force plates in military training scenarios. In military training school environments, there is often no equivalent to the pre-season training interval, where an individual is assessed and trained to strengthen vulnerabilities or train for specific tasks required by their sport. Also, training loads are not quantified and muscle skeletal injury, while common, is not uniformly evaluated and treated to return the service member to the training school in the same way that an athlete would be qualified to return to play. The skills and characteristics that force plate technology assessments are evaluating may or may not be applicable to the skills that are taught in military training. Military training schools train physical skills and mindset against a greater variety of tasks that often need to be performed on land and water in austere conditions that are not defined by a game clock. Knowledge of how technologies, like force plate assessments, add value to military training objectives is needed. These training objectives include aerobic and endurance performance that has previously been shown to be measurable in athletic and military populations using vertical jump kinetics as measured with force plates [4–6]. In addition, measures obtained by force plate assessments are exposed to proprietary software and results are reported to the user across categories meant to reflect biomechanical, muscle skeletal and proprioceptive traits such as strength, balance, and explosiveness. These measures are compared to historical data, obtained in elite and other athletic populations, and underlie proprietary predictive training recommendations to better inform training or recovery. The validity of the measures for informing military preparedness, risk of injury or recovery has not been as well studied [3, 7–10].

In this study, we obtained baseline and serial force plate measures to better understand fitness levels and load-over-time in Marines entering a rigorous training program at Reconnaissance Training Company (RTC, School of Infantry-West, Camp Pendleton, CA). Failure to complete RTC training is due to both mental and physical reasons and is often voluntary [11]. Marines entering the course have a wide range of experience, overall fitness and fitness for the tasks required [12]. We used logistic regression analysis with graduation as the outcome variable to determine whether force plate scores predicted graduation from RTAP as well as an additional repeated measures analysis to characterize the individual variability of force plate metrics over time. Our intent was to determine if force plate measures could provide individualized information that would be predictive of the success or failure of an individual in the training program as well as to better understand the overall training load on individual Marines as they progressed through the 90-day program of instruction. Our previous work at RTC examined predictors of success in the course and we reported that older age at entry, time in the Marine Corps prior to entering training at RTC, optimistic mindset, a history of strong aquatic skills and some, but not all, physical fitness test scores (PFT) as positively correlated with success in the training program [11, 13].

## Methods

### Reconnaissance Training Course (RTC)

The RTC course structure has been previously described and includes an initial 25-day training (RTAP, Reconnaissance Training and Assessment Program) followed by a 12-week Basic Reconnaissance Training Course (BRC). Historically, attrition in the initial RTAP phase typically ranges between 45% and 81% of trainees and success in BRC ranges from 50-85% [11, 13].

Two consecutive classes of Marines entering RTAP (6/8/21 to 8/25/21) were offered study enrollment. Subjects signed informed consent for participation and completed a demographic survey. Enrollment included 52 Marines from the first RTAP class and 60 Marines from the second RTAP class, totaling 112 Marines. Study participants who successfully completed RTAP were followed with four additional serial force plate measures administered every 30-days and at the end of the BRC course (Table 1). After successful graduation, the initial RTAP class waited 30 days prior to enrolling in BRC and were merged with the second RTAP and class graduates. Their duties in the waiting interval included participation in land and water Reconnaissance training exercises (Marines Awaiting Reconnaissance Training, MART). The second RTAP class entered BRC 5 days after course completion.

**Table 1:** Force plate assessments administered across training time points

**Observation** 30-Day Intervals	**Course Progress**
1	Baseline (pre-RTAP)
2	Post-RTAP, day 30
3	Pre-BRC
4	Mid-BRC
5	Post-BRC

The demographics of the study subjects is shown in Table 2.

**Table 2:** Demographics of study subjects

	**Overall (*N*=112)**
**Age (years)**	
Mean (SD)	21.8 (3.31)
Median [Min, Max]	21.0 [18.0, 32.0]
**Height (cm)**	
Mean (SD)	179 (5.81)
Median [Min, Max]	178 [165, 191]
**Weight (kg)**	
Mean (SD)	80.4 (9.11)
Median [Min, Max]	81.2 [61.2, 109]
**BMI**^a^	
Mean (SD)	25.1 (2.26)
Median [Min, Max]	25.1 [19.4, 31.7]
**Time in Military (months)**	
Mean (SD)	36.8 (26.5)
Median [Min, Max]	24.0 [15.0, 152]
**Rank**	
Captain	2 (1.8%)
Corporal	9 (8.0%)
First Lieutenant	7 (6.3%)
Lance Corporal	23 (20.5%)
Private	7 (6.3%)
Private First Class	56 (50.0%)
Sergeant	7 (6.3%)
Staff Sergeant	1 (0.9%)

^a^*BMI* Body Mass Index, weight (kg) / height (m)^2^

RTC Marine trainees undergo Physical Fitness Tests (PFT’s) as part of the RTAP standards assessment. These include pull-ups, sit-ups, push-ups, a timed unweighted 3-mile run, and 500m swim. A timed land navigation coordinate identification exercise is an additional requirement to graduate RTAP.

### Sparta science force plate scores and assessments

Sparta Science reports force plate measures based on proprietary models and analysis derived from kinetic forces measured on the force plate from a vertical jump. Individual scores are compared to normative values (NV) derived from prior scores on the force plate [10]. The jump data is reported as an overall movement score (Sparta Score, NV, mean = 81.8%, SD = 5.9%), that is a percentage value. Additional scores termed Load (eccentric rate of force development, NV, mean = 50, SD = 10), Explode (relative concentric force development from upward force transfer, NV, mean = 50, SD = 10) and Drive (concentric relative impulse from impact of initial force applied, mean = 50, SD = 10) scores are reported on a scale of 0-100 for each jump. A risk of injury score (MSK Health score, NV, mean = 53.3, SD = 7.7) is also provided.

Additional assessments of balance and core stability can be measured from the force plate but do not drive the calculation of force plate scores above. Training recommendations are provided by Sparta Science, based on all measures, and these were not analyzed or utilized in this study.

All trainees underwent a physical trainer-supervised warm-up prior to a force plate assessment that included 5-10 lateral and reverse lunges, and demonstration and practice of the correct body position and motions required to perform a countermovement vertical jump on a force plate. A single assessor witnessed the force plate assessments that included five countermovement vertical jumps per trainee with a rest interval of 10 seconds, as indicated by the Sparta Science. This was repeated for each consecutive force plate assessment.

### Statistical analysis

Independent *t* tests were conducted on continuous variables to compare those who graduated versus those who dropped RTAP. Multiple logistic regression analyses were conducted with graduation as the outcome variable as well as to determine whether Fitness predictors alone, Sparta scores alone, and combined Fitness + Sparta scores predicted graduation from RTAP. ROC curves were computed to characterize the predictive ability of Sparta scores only, fitness scores only, and fitness + Sparta scores. For the repeated measures analysis, within-subjects confidence intervals were computed to remove between-subject variability across the five repeated force plate assessments [13].

## Results

### Graduation success

A total of 49 (44%) trainees entering RTAP successfully completed the course. Of these 38 (78%) graduated from BRC. The majority of the RTAP training failures were due to failure to complete the land navigation exercise (19/66, 34.7%) or voluntary withdrawals (Drops on Request [DOR], 18/66, 27.2%). The remainder of RTAP non-completions were due to failure to meet PFT standards (11/66, 16.7%), medical illness or injury (8/66, 12.1%), safety concerns (5/66, 7.6%) or administrative training (2/66, 3.0%) withdrawals. The majority of BRC graduation failures were due to navigation or aquatic training failures (6/11, 54.4%%), medical illness or injury (3/11, 2.7%) or DOR’s (2/11, 1.8%). Graduation rates did not differ between trainee groups 1 or 2 for RTAP and BRC.

### Comparison of RTAP graduates and non-graduates by baseline performance

There were no significant differences in age, height, weight, or BMI between those trainees who did and did not graduate RTAP. Table 3 examines the relationship between baseline (initial) Physical Fitness Test scores (pull-ups, sit-ups, push-ups, 3-mile run, and 500m swim) and baseline force plate scores in relation to RTAP course graduation success. Better performance on baseline PFT tests were significantly related to graduation rates. For the force plate measures, overall baseline Sparta Score, but not other scores, was weakly predictive of RTAP graduation.

**Table 3:** Comparison of Baseline PFT Performance and Sparta Scores: Graduates to Non-Graduates

PFT Performance	Graduates (*N*=49)	Non-Graduates (*N*=63)	*P* Value
**Pull-ups (reps)**			
Mean (SD)	18.1 (3.68)	15.7 (5.12)	<.005
Median [Min, Max]	18.0 [11.0, 24.0]	17.0 [0, 25.0]	
**Sit-ups (reps)**			
Mean (SD)	102 (11.6)	95.0 (15.3)	<.01
Median [Min, Max]	102 [79.0, 125]	94.0 [63.0, 133]	
**Push-ups (reps)**			
Mean (SD)	81.6 (14.5)	70.7 (15.2)	<.001
Median [Min, Max]	82.0 [56.0, 108]	70.0 [35.0, 116]	
**3-mile run (min)**			
Mean (SD)	19.6 (1.12)	21.0 (1.52)	<.001
Median [Min, Max]	19.0 [18.0, 22.0]	21.0 [18.0, 26.0]	
**500m swim (min)**			
Mean (SD)	14.0 (1.46)	15.4 (2.32)	<.001
Median [Min, Max]	14.0 [10.0, 20.0]	15.0 [12.0, 23.0]	
Sparta Scores	Graduates (*N*=49)	Non-Graduates (*N*=63)	*P* Value
**Sparta Score**			
Mean (SD)	78.2 (4.20)	76.8 (3.25)	.049
Median [Min, Max]	78.0 [67.0, 86.0]	76.0 [71.0, 85.0]	
**Load**			
Mean (SD)	43.0 (8.12)	41.8 (7.87)	.45
Median [Min, Max]	42.1 [33.3, 71.3]	39.9 [29.9, 66.7]	
**Explode**			
Mean (SD)	38.8 (7.37)	37.4 (6.75)	.32
Median [Min, Max]	39.3 [20.7, 60.7]	36.9 [27.8, 55.3]	
**Drive**			
Mean (SD)	53.4 (9.27)	52.1 (9.94)	.5
Median [Min, Max]	52.9 [34.5, 72.1]	52.5 [30.0, 70.2]	
**MSK Health**			
Mean (SD)	59.4 (4.71)	58.5 (5.74)	.35
Median [Min, Max]	59.0 [49.0, 69.0]	57.0 [46.0, 71.0]	

### Subgroup analysis for effects of baseline physical fitness tests and Sparta scores on RTAP graduation

**Fig. 1:**
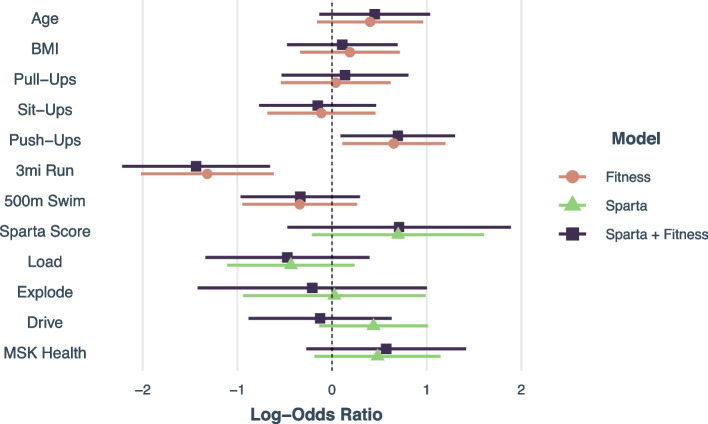
Hazard Ratio Associations with Graduation

As compared to PFT performance, the addition of Sparta scores added little to the predictive power of the PFT assessments to predict graduation rates. The 3-mile run, and swim assessments were the only measures consistently below 1.0, indicating a significant association with higher graduation rates (Fig. 1). PFT scores had the highest predictive accuracy of 78.9% ± 12.5% compared to Sparta Scores or PFT scores + Sparta Scores (64.8 ±11.2,77.5% ± 9.0%).

**Fig. 2:**
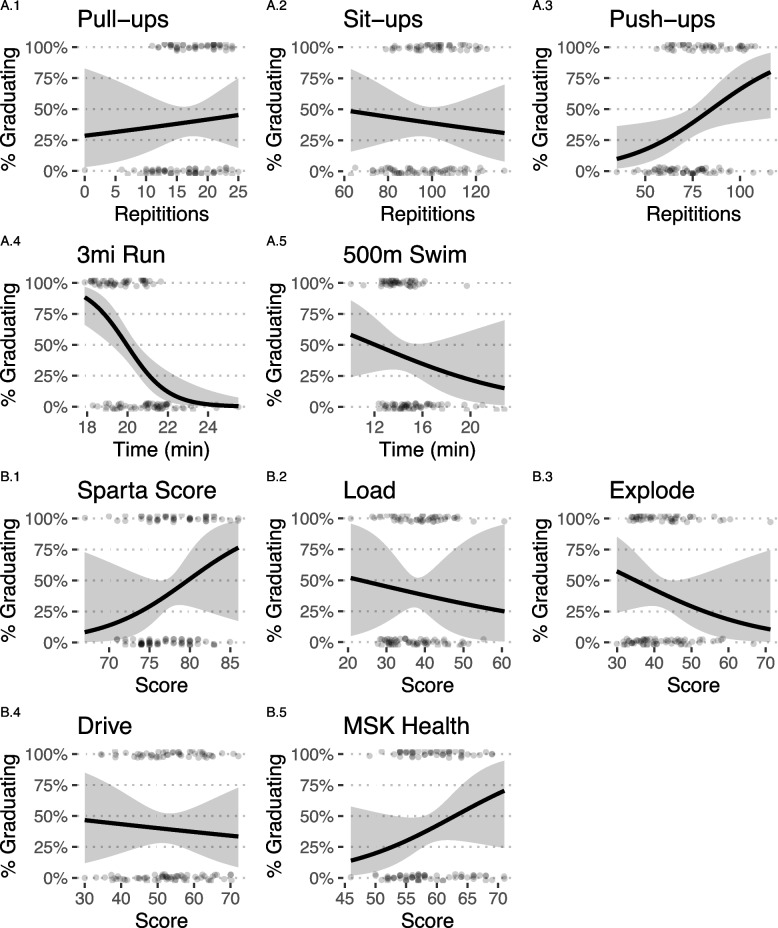
Effect plots of 3mi run time, push-up repetitions, Sparta Score, and MSK Health Score predictors

Figure 2 depicts effect plots showing the predicted probability (CI 95%) of graduation across values for each independent variable. The black line represents the predicted probability of graduation across levels of each predictor variable while holding all other variables constant. The shaded gray area represents the confidence interval range. Sparta scores (Load, Explode, Drive) show wide confidence interval bands, with poor predictive accuracy. For example, in the timed 3-mile run, trainees with times of 18 minutes 50 seconds have a 75% predicted probability of graduation with all other variables held constant. Trainees with 3-mile run times of 20 minutes are predicted to have a 50% chance of graduation, while trainees with 3-mile run times of 21 minutes 5 seconds are predicted to have a 25% predicted probability of graduation.

### Serial force plate measures in graduates

**Fig. 3:**
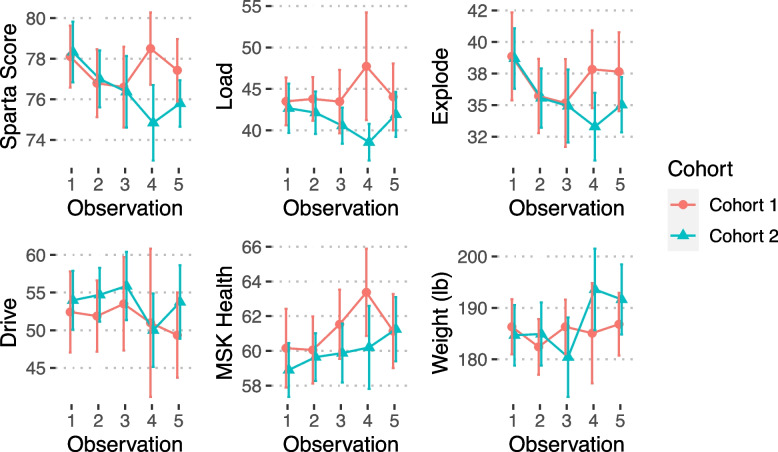
Serial Sparta Scores Trainee Group 1 and 2

**Fig. 4:**
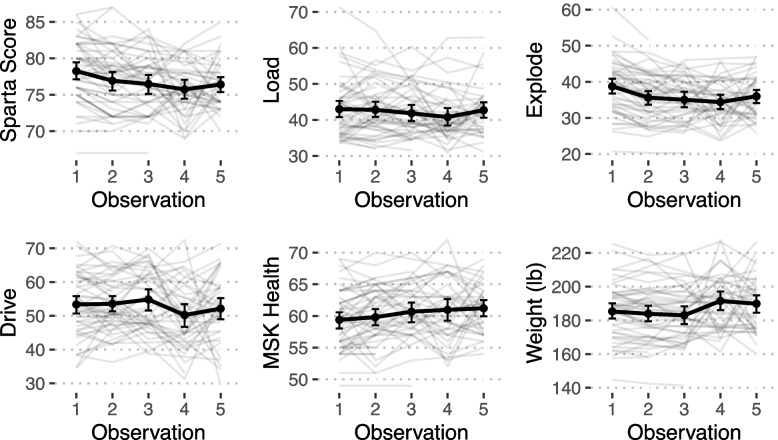
Serial Sparta Scores, trainee groups combined

Figures 3 and 4 demonstrate serial force plate scores obtained every 30-days in those trainees that progressed successfully through the course. In Fig. 3, trainee group 1 scores are shown in red. These are the trainees that had 30-days off between the initial RTAP course and the 60-day BRC course. Scores in green are those of trainee group 2 (these RTAP trainees went directly from RTAP to BRC). Observation 3-4 reflects the time interval from the end of RTAP through the first month of BRC. A clear difference in Sparta scores is seen between the two groups and trainee group 1 demonstrates better scores for this time period. When the two trainee groups are not separated (Fig. 4, black bold line), most Sparta scores decline in this interval and recover toward the last month of training, but not back to the baseline obtained in the beginning of the course. Interestingly, there is a weight gain observed at the end of the course. There was significant intra- and inter-individual variability across each force plate measure (Fig. 4, light gray lines).

## Discussion

We have previously reported that there is value in leveraging connected technology to gain insights into Marine performance in training [11]. We accurately measured mental and physical attributes of RTC trainees with customized software and digitally connected wearable sensors (Apple Watch) continuously throughout training in land and water. This dataset has allowed the identification of novel and early predictors of failure to complete the course. We have also identified how pre military experiences, including prior athletic habits, family background and motivation to become a Reconnaissance Marine are predictive of successful RTC graduation [12].

In the case of the force plate technology that had been purchased for the School of Infantry-West, at Camp Pendleton, we sought to determine if we could find a military use case for the technology within the training environment. Force plate assessments have the potential to add to the knowledge base by providing an efficient capability to understand an individual’s muscle skeletal performance and kinetics. This could enable the development of individual Marine trainee profiles indicative of fitness, balance, and strength [1]. Force plate assessments have identified the vertical jump (VJ) to assess and develop risk profiles for injury through identification of dynamic weaknesses, and this data has been shown to be reproducible [7–9]. Some studies have shown the utility of using the VJ measured via force plate analysis to be a viable option to quantify rates of muscular fatigue during an endurance activity like an ultramarathon, and Sparta Science variables have been shown to be reliable in military populations, despite the fact that military physical training movements are more focused on the traditional aerobic/endurance model than the more explosive movement patterns (e.g. plyometrics) of other types of athletes measured by Sparta Science products [4–6]. In this study, however, despite providing training and having a consistent repeatable protocol with physical trainer observation, there was significant intra- and inter- individual variability, most likely reflecting the fact that Marine trainees are not familiar with, nor do they have prior training in technique related to generating maximum forces throughout the force plate assessment. This limits the ability to draw conclusions related to the general fitness of a training group cohort. This is consistent with our finding that baseline values of most Sparta scores in Marine trainees are lower than Sparta reported normative values. This variability also renders the goal of creating a load-over-time score less likely to be achieved with force plate technology measures alone in military personnel [1].

Force plate measures have been associated with prediction of injury vulnerability in elite athletes, as well as reductions in health care utilization and costs, independent of actual reductions in injury. This suggests that force plate assessments and subsequent training to correct or strengthen deficiencies can result in less severe injuries [7–9]. In a recent study conducted in over 800 Air Force Trainees, force plate assessment did not predict risk of military training injury, suggesting that this military population or training is not comparable to elite athletic training injury risk. Characterizing high and low risk groups for injury using force plate and other kinetic measurement technologies has been recently reported in other Marine training courses, which may prove useful if training regimens can be implemented in those at risk [14]. In this study, we were less interested in predicting injury because injury is not as significant a source of attrition within the RTC training as DOR’s or academic issues. Unlike an elite athletic environment that can take advantage of a pre-season or has committed in-season strength and conditioning resources, the nature of most military schedules and training permits very little time and is under-resourced to provide an individual the ability to address deficiencies identified by force plate measures through directed training. The study found that a single force plate measure is not predictive of graduation due to physical or psychological degradation resulting in attrition. Our data also suggest that serial force plate assessments do not contribute novel information to help identify causes of attrition throughout a 90-day training course. This suggests that dynamic musculoskeletal assessments do not reflect or equate to standard PFT tests performed in land and water.

Serial force plate assessments do have the potential to identify and quantitate how Marine RTC training impacts musculoskeletal health and performance over time. Our findings that serial assessments vary depending on the loads introduced by the training is not surprising, but it offers little new data in terms of insights that might direct training optimization. The finding that trainees who had a month off between classes had improved force generation when re-entering training is interesting and suggests more recovery in these Marines compared to those entering the second set of classes immediately after completing the first. The fact that this difference did not predict an advantage in graduation success renders it less important. In this study, when the two groups were combined students experienced recovery in the last month of the course as measured by force plate score improvements. This finding may be indicative of the fact that the training in that interval favors aquatic training, which entails lower musculoskeletal loads than land training. Another confounding issue in interpreting vertical jump force plate measures is the lack of normative values in military populations and the wide variance of scores in the published literature in athletic and military populations. One study of a diverse group of Division 1 collegiate athletes identified both increases and decreases in Load, Explode, and Drive as predictive of an ACL injury. This makes it difficult to know if increases or decreases in scores reflect degradation during training or augmentation [9]. Similarly, a study in over 500 professional baseball pitchers found that force plate measures could predict elbow but not shoulder injury and that specific force plate profiles of elbow injury risk consisted of both increases and decreases in force plate generation [7]. Finally, our study findings suggest that further work needs to be done to understand how to benefit military trainees with force plate technology. Rehabilitation from injury once it occurs and identifying kinetic traits that can be augmented prior to training or after rigorous training merit further study in military populations.

### Limitations

We did not achieve 100% enrollment in the study (70.4% of trainees offered participation in the study chose to enroll) and serial measures could not be obtained in all study enrollees who successfully completed the course. Thus, repeated assessments were not used to predict the main outcome because it was confounded with early drops from course. It may be that data from non-enrolled trainees would have led to different conclusions. Despite instructing trainees on proper force plate technique, there was significant intra-individual variability in measures for each force plate assessment. This lack of reliability limits any conclusions related to force plate assessment values obtained over time. Additionally, the time-gap between groups (one group started 30-days after their previous training program while the other started immediately) may have impacted the reliability of the group comparisons.

## Data Availability

The datasets generated during and/or analyzed during the current study are available from the corresponding author on reasonable request.
